# Customer Context Analysis in Shopping Malls: A Method Combining Semantic Behavior and Indoor Positioning Using a Smartphone

**DOI:** 10.3390/s25030649

**Published:** 2025-01-22

**Authors:** Ye Tian, Yanlei Gu, Qianwen Lu, Shunsuke Kamijo

**Affiliations:** 1 Institute of Industrial Science, The University of Tokyo, Tokyo 153-8505, Japan; lu-qianwen17@g.ecc.u-tokyo.ac.jp (Q.L.); kamijo@iis.u-tokyo.ac.jp (S.K.); 2 Graduate School of Advanced Science and Engineering, Hiroshima University, Higashi-Hiroshima 739-8525, Japan; guyanlei@hiroshima-u.ac.jp

**Keywords:** customer context analysis, human behavior recognition, pedestrian dead reckoning, indoor positioning

## Abstract

Customer context analysis (CCA) in brick-and-mortar shopping malls can support decision makers’ marketing decisions by providing them with information about customer interest and purchases from merchants. It makes offline CCA an important topic in marketing. In order to analyze customer context, it is necessary to analyze customer behavior, as well as to obtain the customer’s location, and we propose an analysis system for customer context based on these two aspects. For customer behavior, we use a modeling approach based on the time-frequency domain, while separately identifying movement-related behaviors (MB) and semantic-related behaviors (SB), where MB are used to assist in localization and the positioning result are used to assist semantic-related behavior recognition, further realizing CCA generation. For customer locations, we use a deep-learning-based pedestrian dead reckoning (DPDR) method combined with a node map to achieve store-level pedestrian autonomous positioning, where the DPDR is assisted by simple behaviors.

## 1. Introduction

Analyzing shopping behavior is crucial for understanding the effectiveness of marketing and sales efforts. Through in-depth shopping behavior analysis, operators of large shopping centers can capture customer preferences, adjust marketing strategies, or modify the layout of the stores within the mall. However, there is still a lack of effective and convenient methods to comprehensively identify customer behavior. In physical stores, the most readily available shopping behavior data are customer purchase records, but these are difficult to link to individual customers, so they can only be used for general user group statistics, and do not include information from before the purchase, such as which stores the customers browsed in the shopping center, which stores they were interested in, and how much time they spent in different stores. Therefore, it is crucial to explore new methods for capturing customer behavior in physical stores.

Preliminary research attempts to capture customer trajectories and routes in large shopping centers by using sensors associated with users, such as smartphones (e.g., inertial sensors, Wi-Fi, etc.), aiming to determine the time customers spend in different stores based on their trajectories, which can then be used as an indicator of user preferences [1,2]. However, this location-based approach struggles to capture sufficient semantic information. Subsequent studies have tried using densely deployed surveillance cameras [3] or radio frequency identification [4,5,6,7,8] technology to monitor shopping behavior, providing rich semantic content. However, these vision and RFID-based methods are heavily reliant on preinstalled environmental sensors, which restricts the application of customer behavior capture to specific retail stores, making it difficult to scale up across large shopping centers.

Unlike the methods mentioned above, we aim to develop a rich semantic customer context analysis method based on smartphone sensors. This approach offers several advantages: (1) It allows linking to specific customers while maintaining their anonymity; (2) It does not rely on preinstalled ambient sensors, making it more suitable for application in large shopping centers; (3) It not only relies on trajectories but also considers complex activities and the semantics of their interactions with the environment.

The main contribution of this paper can be summarized as follows. (1) We develop a customer context analysis method, which is deeply integrated by an indoor autonomous positioning module and a behavior recognition module. (2) We construct behavior-based DPDR method to realize indoor autonomous positioning. The behaviors will assist in estimating velocity, and a map-matching method will assist determine the absolute position of the customer. (3) We conduct extensive well-designed experiments in one open-source dataset, i.e., the RIDI [9] dataset. for indoor positioning and one self-collected dataset for customer lifelogging. Furthermore, we conduct an entire experiment in a typical shopping mall scenario, a shopping mall in the Tokyo area, to evaluate the whole system.

## 2. Related Work

### 2.1. Human Behavior Recognition (HAR)

We are attempting a smartphone-based approach to capture more complex customer semantics combining with location, rather than just location-based data. The development of activity recognition technologies in recent years supports this effort. Refs. [10,11,12,13] have used deep learning methods to recognize movement-related behaviors, and we believe that such activities can be beneficial for assisting pedestrian localization. Refs. [14,15,16,17,18] have proposed the recognition of daily activities, enhancing the semantic understanding of activities. Previous work [19] has distinguished between simple and complex activities, further enriching the semantics of the activities.

### 2.2. Indoor Positioning and Pedestrian Dead Reckoning (PDR)

In the customer context analysis (CCA) of large shopping centers, although user behavior provides rich semantics, location information remains indispensable. The development of indoor positioning technologies supports this possibility, such as methods based on wireless signals (e.g., WiFi [20], 5G [21] Bluetooth [22]) and vision-based methods [23,24]. However, these methods also have the significant drawback of heavily relying on preinstalled environmental sensors. To avoid this issue, we choose to use a smartphone-based inertial sensor positioning solution.

PDR is a positioning method for pedestrians that uses human body information as constraints to estimate location by calculating the pedestrian’s stride length and heading. Past work has made significant efforts in velocity estimation for PDR. Traditional methods usually use empirical models [25] to estimate velocity, but in recent years, velocity estimation methods based on deep learning have been developed. Ref. [26] proposed using a denoising autoencoder to estimate velocity, addressing issues of sensor bias and noise. Refs. [27,28] proposed a model combining LSTM and denoising autoencoder to further improve the accuracy of velocity estimation. Ref. [29] introduced using the ResNet50 framework to estimate velocity, considering the impact of different handheld modes on estimation results. Therefore, the handheld mode is preidentified, and then stride estimators are trained separately for different modes.

### 2.3. Customer Context Analysis

Some existing studies have made efforts toward different types of CCA, which can be broadly categorized into two approaches: user-bound sensor-based methods and environment sensor-based methods. Refs. [1,2] used smartphones to track customers’ locations, using position as the sole semantic source to determine which stores customers have visited and how long they stayed in those stores. Ref. [30] proposed using a combination of sensors from smartwatches/smartphones, along with environmental anchors for precise hand positioning, to recognize actions such as “picking up an item” with shelf-level accuracy.

The environment sensor-based approach has received more attention in the past. Ref. [30] used Bluetooth anchors to achieve fine-grained, shelf-level positioning. Ref. [4] utilized RFID tags to capture shelf-level customer semantic Ref. [3] employed surveillance cameras to monitor customer behavior. Ref. [31] integrated multiple ambient sensors to create an unmanned store. Although the environment sensor-based methods can provide substore-level, more detailed customer semantics, the trade-off is the need for a large number of preinstalled environment sensors, which usually limits the application scenarios to specific stores rather than large shopping centers.

## 3. Problem Definition

### 3.1. Customer Context Analysis (CCA)

We defined the content of the consumer context analysis task. We set the task scenario in a large shopping mall, with the goal of understanding the behavior of consumers within the mall. The task primarily includes (a) the shops where customers stay and the duration of their stay in each shop and (b) the behaviors of customers in different shops and the duration of each behavior. We believe that this definition can describe consumers’ behavior patterns and their level of interest in different shops.

### 3.2. Semantic Location Recognition and Dwell-Time Estimation

We aim to define the context indoor positioning task within the CCA framework. In general indoor positioning, absolute or relative positioning errors are used to evaluate accuracy, with the error typically measured in meters or decimeters. However, in the CCA task, we focus more on shop-level positioning accuracy. We have created a semantic floor node map for a large shopping mall, where semantic nodes include shops, shop entrances, intersections, elevators, and escalators. The goal of CCA indoor positioning is to achieve node-level positioning results.

### 3.3. Behavior Definition and Analysis

In this paper, we categorize user behaviors into two types: movement-related behaviors (MBs) and semantic-related behaviors (SBs), where MBs represent fundamental physical actions that constitute human behavior, describing basic, universally shared walking patterns, and SBs, on the other hand, are complex activities derived from random combinations of MBs, which, when further combined with environmental semantics, denote semantic activities that characterize shoppers’ behaviors within large shopping malls. Specifically, MB include: standing still, walking straight, and walking laterally. These behaviors are relatively simple, involving the periodic repetition of basic subactions. On the other hand, semantic-related activities are more complex and are typically characterized by non-periodic random combinations of basic actions. We summarize and distinguish these behaviors in Table 1.

In our task, semantics and environment are tightly coupled. We believe that location information can provide additional constraints on behavior. As shown in Table 1, we generally assume that the behavior of selecting items only occurs inside stores, and that floor changes only occur at elevator/escalator nodes.

## 4. Proposed Method

**Figure 1:**
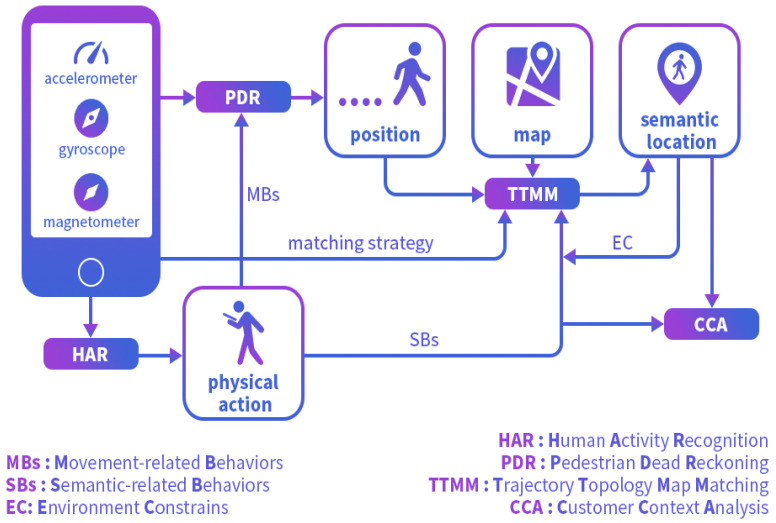
Overview of our CCA method.

The proposed method is divided into two main parts: the indoor semantic positioning module and the semantic behavior recognition module. As shown in Figure 1, these two modules are closely integrated. First, semantic localization is performed, where velocity estimation is constrained by MBs based on multi-task constraints. The estimated velocity is then combined with heading estimation to obtain the PDR trajectory. Using a Hidden Markov Model (HMM), the PDR trajectory is mapped to shop-level locations, allowing us to determine the specific store where the customer is located. Floor estimation is considered part of the localization module, but it actually uses information from behavior semantics. We use elevator/escalator behaviors to trigger floor changes.

### 4.1. MB Recognition

**Figure 2:**
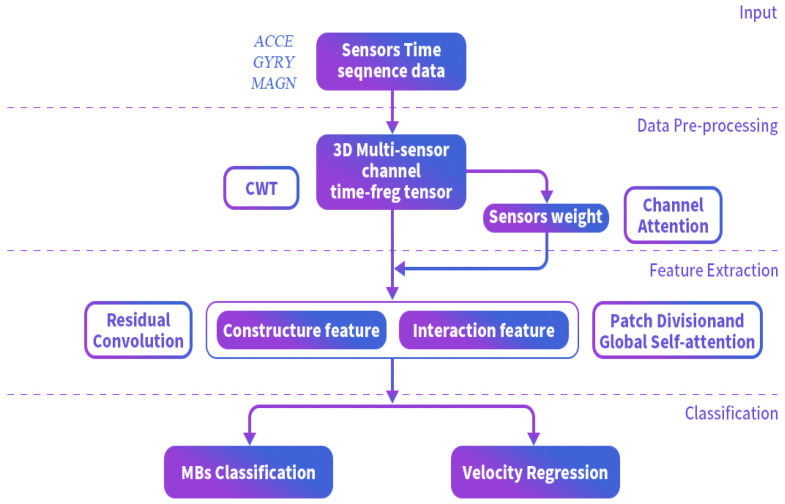
The structure of our multi-task velocity estimation method.

In this section, we will introduce the semantic positioning approach in detail. We propose using velocity estimation instead of stride length estimation to modify the traditional PDR. This approach helps avoid errors introduced by the pedometer. First, for velocity estimation based on multi-task constraints, we incorporate the feature extractor from our previous work [32]. This feature extractor takes the time-frequency spectrum of short sliding windows of sensor data (typically 1 s) as input, uses a CNN to extract local features, and then applies parallel self-attention to compute global features. This method achieved high-accuracy recognition of MB, and is shown in Figure 2.

We introduced a multi-task loss function to guide the model in simultaneously estimating both behavior and velocity. These two estimation tasks share features, which allows the velocity estimation to take behavior label information into account. This is based on our assumption that a pedestrian’s velocity is coupled with their MBs. This loss function is composed of the cross-entropy loss for behavior label classification and the mean squared error (MSE) loss for velocity regression, as shown in Equation (1):

(1) $$Loss=-\sum_{i=1}^{n}p\left(x_{i}\right)\log\left(q\left(x_{i}\right)\right)+\frac{1}{n}\sum_{i=1}^{n}{\left(y_{i}-h\left(x_{i}\right)\right)}^{2}$$

where the first summation term is the cross-entropy loss function and the second summation term is the MSE loss function.

### 4.2. Pedestrian Dead Reckoning

In the previous subsection, we obtained the velocity estimation. In this section, we can combine it with direction estimation to perform PDR, and thereby, obtain the position as Equation (2):

(2) $$p_{i}=p_{i-1}+L_{p}\left[\cos\psi_{i},\sin\psi_{i}\right]$$

where $p_{i}=\left[x_{i},y_{i}\right]$ denotes the 2d position of time step *i*. Each time the algorithm starts, an attitude initialization process is performed, during which the initial heading, pitch, and roll angles are obtained. We use the map’s semantics to acquire the initial heading angle, setting the starting point of the customer’s trajectory at the elevator/escalator or the shopping mall entrance, where the initial heading angle can be easily determined. At the same time, the horizontal leveling matrix of the accelerometer is used to align the roll and pitch angles, a process that requires the sensors to remain stationary for several seconds. After obtaining the initial attitude, the heading angle can be estimated in real time during the subsequent process.

For real-time direction estimation, we first use a static correction method to coarsely correct the gyroscope bias [33], which can be performed simultaneously during the altitude initialization process. In our method, only the accelerometer and the topological map are used as reference for heading estimation, without relying on magnetometer readings. This is because the magnetometer has been proven to be ineffective for observing geomagnetic north in indoor environments [34]. In addition to the accelerometer constraints, when outside of shops, we also use map nodes as pseudo-constraints for heading estimation. We assume that the heading of the user in the corridor outside shops should be clear, meaning that the heading remains constant between two nodes. This pseudo-constraint is defined as the direction vector from the current node to the next possible node. We perform turn detection at a given node: if no significant turn is detected, we choose the direction of the node directly in front of the user as the reference. If a significant turn is detected, we select the node direction that is closer to the heading calculated by the gyroscope as the reference.

### 4.3. Trajectory Matching with a Topology Map

In our task, the classic Viterbi algorithm is used to obtain semantic location with node-level accuracy. We formulate map matching as a HMM. The position $p_{i-1}$ is recorded after the map is successfully matched at step i−1. The PDR system accumulates position updates at a constant frequency (1 Hz, e.g.), and we calculate the transition probability using $p_{i-1}$ and the accumulated position change $d_{i-1}$. When the Transit Probability (TP) exceeds a threshold T, the match is considered successful, and the position is updated to $p_{i}$.

**Figure 3:**
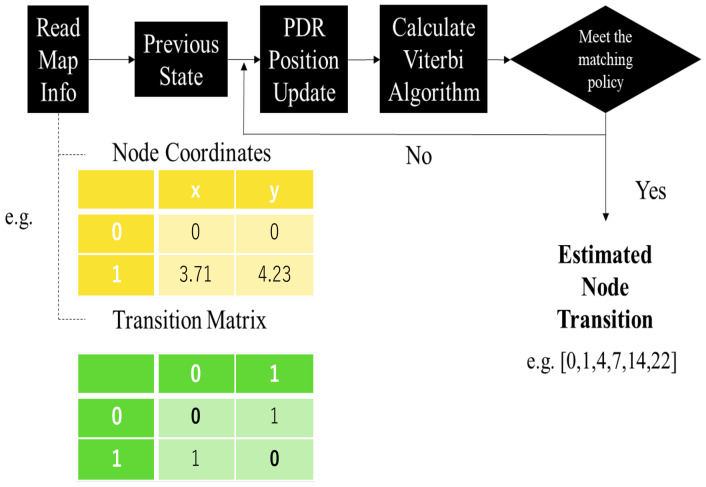
The strategy of the map matching module.

When creating the node map, the connections/disconnections between nodes become clear. In this study, two types of information are stored on the smartphone as the node map, where one is the absolute coordinates of the nodes and the other is the connections/disconnections between nodes. In Figure 3, the process of creating the node map is explained. The connections/disconnections between nodes are represented as a two-dimensional array of node count by edge count. If a user cannot move from one node to another, a ‘1’ is inserted; otherwise, a ‘0’ is inserted. By completing this matrix, we define TP.

The probability distribution calculated from the difference between the node coordinates and the observed coordinates obtained through turn detection is actually decomposed into distance and angle, which also better aligns with the PDR calculation process. In other words, the distance and angle between nodes are compared with the distance and angle between $p_{k}$ and $p_{i-1}$. First, the distances and angles between nodes can be precomputed. Each time the PDR is updated, the distance and angle between the observed coordinates are calculated. When each time PDR updates, for all nodes, the distance difference $dif_{dis}$ between the node and the observed coordinates, as well as the angle difference $dif_{ang}$ between the node angles and the observed coordinates, are computed. Using these differences, the probability distribution is then calculated as follows:

(3) $$\begin{array}{c} P\left(dif_{dis}\right)=\exp\left(-{\left(\frac{dif_{dis}}{\sigma_{dis}}\right)}^{2}\right)\\ P\left(dif_{ang}\right)=\exp\left(-{\left(\frac{dif_{ang}}{\sigma_{ang}}\right)}^{2}\right) \end{array}$$

where $\sigma_{dis}$ and $\sigma_{ang}$ are the variances of the respective probability distributions. The probability calculated from the product of these probabilities is the probability calculated from the difference between the coordinates of the nodes and the observed coordinates obtained by each time PDR updates. Thus, the emission probability (EP) is expressed as shown below:

(4) $$EP=P\left(dif_{dis}\right)*P\left(dif_{ang}\right)$$

when the EP of a candidate node is greater than that of other nodes and also exceeds the threshold T, the algorithm considers the match successful. At this point, the Viterbi algorithm is used to optimize the whole history node trajectory.

For floor estimation, we use floor-change behaviors to determine the floor. For floor-change (elevator), after detecting elevator behavior, we estimate the number of floors changed by calculating the pressure difference between the start and end times of the behavior. For escalators, the end of a continuous escalator behavior is defined as a single floor change. When the algorithm detects a floor change, the attitude is reinitialized.

### 4.4. Customer Context Analysis

**Figure 4:**
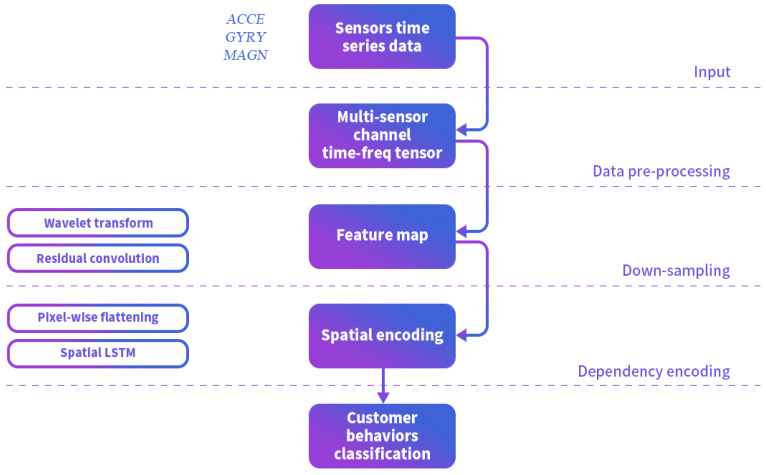
The structure of the SBs’ recognition module.

CCA is defined by SBs that incorporate environmental semantics. In our previous work [19], we also used the time-frequency spectrogram of sensor data as input to refine feature extraction, and proposed a spatial LSTM in the network structure to simultaneously learn sequential combination features in both the time and frequency domains. For complex activity recognition, a longer sliding window is usually used to fully describe SBs. as shown in Figure 4. For complex activity recognition, a longer sliding window is usually used to fully describe a complex activity.

The final CCA result can be obtained by combining the semantic location from map matching with the identified SBs.

**Figure 5:**
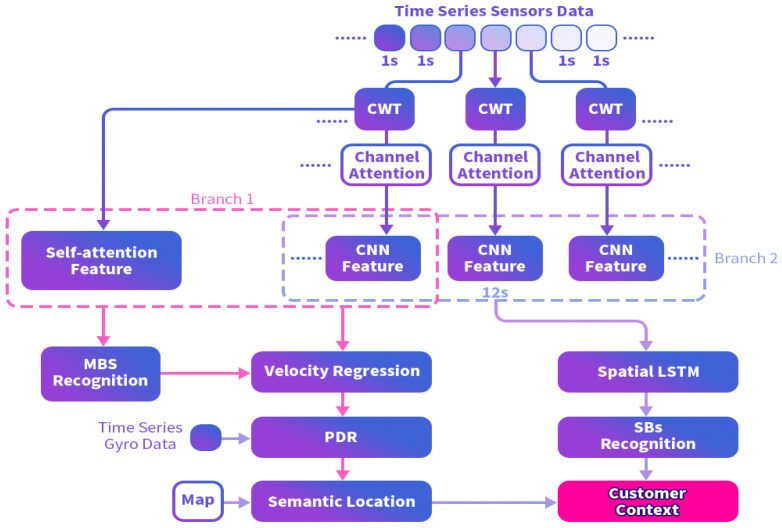
The structure of our customer context analysis.

Specifically, as illustrated in Figure 5, data are input in the form of short time-windows, with each segment transformed into a time-frequency spectrum using the Continuous Wavelet Transform (CWT). In Branch 1, the MBs-Network extracts features from the short time-window data, employing a parallel structure that combines channel attention-CNN and global self-attention. According to Equation (1), the multi-task output layer performs basic MBs prediction and speed regression.

In Branch 2, the CNN features from short time-windows are aggregated into long time-window CNN features. Considering the sequential dependencies of long-range time-frequency features [19], the long time-window CNN features are unrolled pixel-wise and input into an LSTM for complex SBs prediction.

In Branch 1, the velocity regression output is utilized for indoor semantic location inference. Combined with the SBs prediction results from Branch 2, the framework ultimately generates a comprehensive semantic analysis of consumer behavior. We list the parameter setting in detail as Table 2.

## 5. Experiment Result and Analysis

We evaluate our CCA system from several aspects, including customer behavior recognition performance, shop recognition and dwell time estimation performance, as well as the overall performance of the CCA system.

### 5.1. Dataset

We used two datasets for evaluation: the RIDI dataset and the Tokyo dataset. Among them, RIDI is a widely used public dataset for evaluating stride length estimation performance. The RIDI dataset includes accelerometer and gyroscope data from a Tango phone, as well as ground truth positions based on a VIO (Visual-Inertial Odometry) system. We apply time differencing to the ground truth positions to obtain per second velocity labels for supervised training. The RIDI dataset does not include user behavior information but does contain different smartphone carrying modes, such as on the thigh, in hand, and in a bag. This is not entirely aligned with our objectives, but using this dataset allows us to evaluate the scalability of our approach.

We used a self-collected dataset for evaluation: the Tokyo dataset. Since existing methods also do not account for the deep coupling between behavior information and stride length estimation, it is currently difficult to find public datasets that include user behavior information. To address this gap, we propose the Tokyo dataset.

In the Tokyo dataset, we proposed a set CCA behavior estimation training dataset, and a final CCA test set. For the velocity estimation dataset, we collected data in open areas using an iPhone as the data collection device, gathering accelerometer, gyroscope, and geomagnetic data, as well as velocity labels derived from GPS position time differencing. To ensure robustness in training, we also included different smartphone carrying modes as labels in the behavior annotations. All velocity estimation data were segmented into samples using a sliding window with a length of 1 s and a step size of 1 s.

For the CCA behavior estimation dataset, we collected CCA behavior data in various shopping malls (including supermarkets, Uniqlo, etc.). These data include accelerometer, gyroscope, and magnetometer readings along with their corresponding CCA behavior labels. The collected data were segmented into samples using a sliding window of 12 s in length with a 1-s step size. Both the behavior set and velocity set are randomly shuffled, with 80% used as the training set and 20% as the validation set, respectively. The details of the Tokyo dataset are listed in Table 3.

For the CCA test set, we collected three test shopping trajectory by three different subjects in a typical large shopping mall in Tokyo area for the final overall evaluation, which similarly includes accelerometer, gyroscope, and magnetometer data along with their corresponding CCA behavior labels. Additionally, we created a floor node map of the shopping mall, which includes node information for each floor’s shops, elevators, escalators, and intersections.

### 5.2. Evaluation on MB Recognition and Velocity Estimation

We evaluated the performance of the stride length estimation method within the system. First, we compared the proposed method with other methods on the Tokyo dataset. The final evaluation of the method is characterized by the MSE of a single sample of velocity estimation and the overall relative distance error (RDE), as shown in Equation (5):

(5) $$\begin{array}{c} MSE=\sum_{i=1}^{N}\sqrt{{\left(\hat{L_{i}}-L_{i}\right)}^{2}}\\ RDE=\frac{|\hat{dis}-dis|}{dis} \end{array}$$

where $L_{i}$ denotes the i-th stride length and dis denotes the distance of the validation trajectory. Using MSE alone as an evaluation metric is incomplete, as it only represents the absolute value of stride length error, while the error could be either positive or negative. In practical applications, the model typically estimates a complete path, allowing positive and negative errors to cancel each other out, which reduces the overall distance estimation error. This depends on the balance of stride length estimation errors around the true values [26]. We use the RDE over the complete validation set to evaluate this metric. A smaller RDE indicates that the velocity estimation error is more balanced around the true values, leading to better performance in practical use. Compared to other methods, our approach demonstrates higher performance, as shown in Table 4.

It demonstrates that our method can integrate label information from different modalities to improve the accuracy of velocity estimation.

### 5.3. Evaluation on SB Recognition

The recognition method for SBs is evaluated on the self-collected Tokyo dataset, as shown in Table 5.

We redeployed Proposed method and baseline work TPN [37], MSRLSTM [10], DCL [38], ABLSTM [39], PEN [40], and PCL [41] on the Tokyo dataset to evaluate performance in the consumer behavior application scenario. It can be observed that our method provides higher recognition performance for more complex spectral data, and compared to other baselines, it shows a significant performance improvement in recognizing behaviors compared to other behaviors. Among these methods, TPN, MSRLSTM, DCL, ABLSTM, and PEN are all based on raw temporal data features. Specifically, TPN employs a pure 1D-CNN architecture, while ABLSTM enhances performance by using an LSTM architecture with self-attention. DCL combines 1D-CNN and LSTM architectures for further performance improvement. MSRLSTM integrates previous approaches by employing a residual connection-based 1D-CNN + self-attention + LSTM architecture, achieving even higher performance. PEN, on the other hand, combines GoogLeNet and GRU architectures, essentially implementing a CNN + LSTM architecture based on temporal data.

The PCL method, similar to our approach, introduces time-frequency spectra. It processes temporal features with LSTM and time-frequency features with 2D-CNN in parallel, ultimately fusing these features. This method outperforms all the aforementioned approaches but lacks further exploration of time-frequency features.

Our method delves deeper into the time-frequency domain, including long-range features across time-frequency bands and sequential features in the frequency domain. As a result, our method achieves superior performance in recognizing these behaviors.

### 5.4. Evaluation on CCA

We conducted experiments on three test paths from the Tokyo dataset, using the following metrics for evaluation:

(6) $$Acc=\frac{T_{crt}}{T_{total}}$$

where $T_{crt}$ denotes the length of the time samples that are correctly classified throughout the entire time sequence and $T_{total}$ denotes the total length of the entire time sequence.

**Figure 6:**
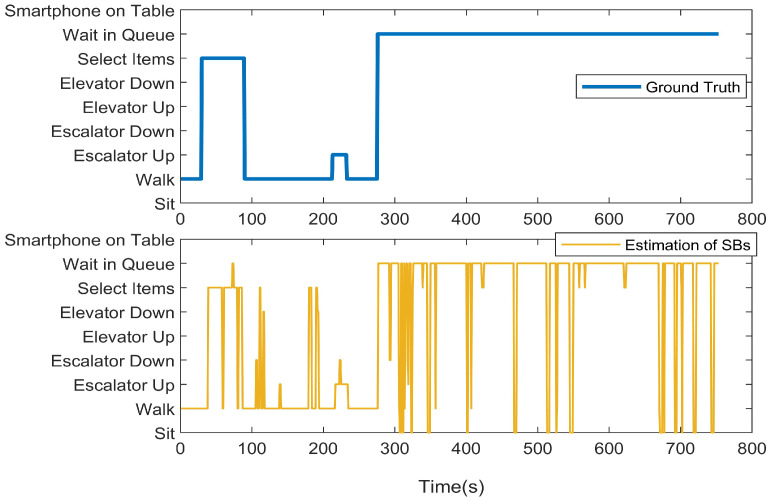
CCA behavior sequence estimation of the test of Trajectory 1.

**Figure 7:**
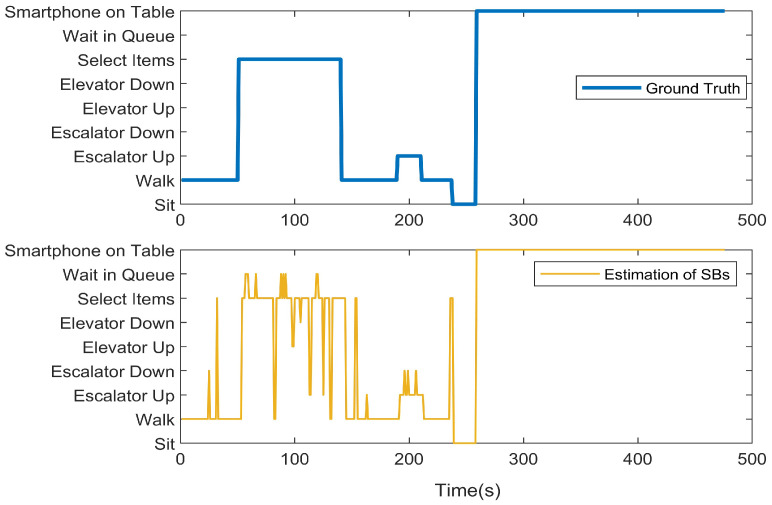
CCA behavior sequence estimation of the test of Trajectory 2.

**Figure 8:**
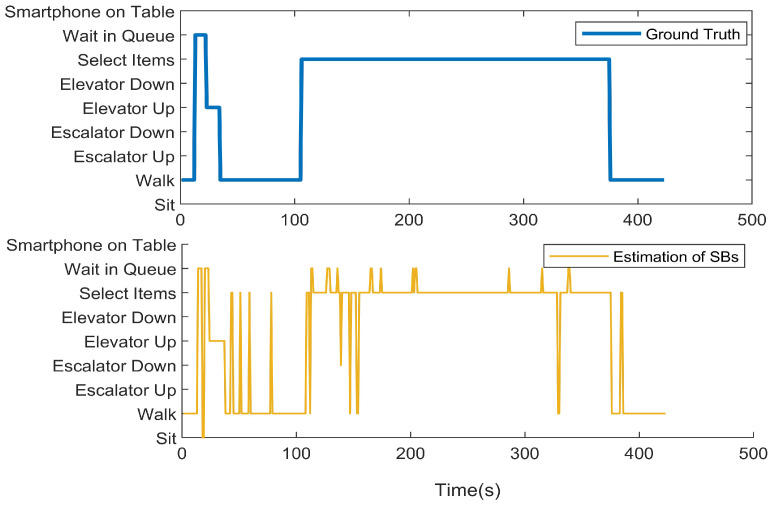
CCA behavior sequence estimation of the test of Trajectory 3.

**Figure 9:**
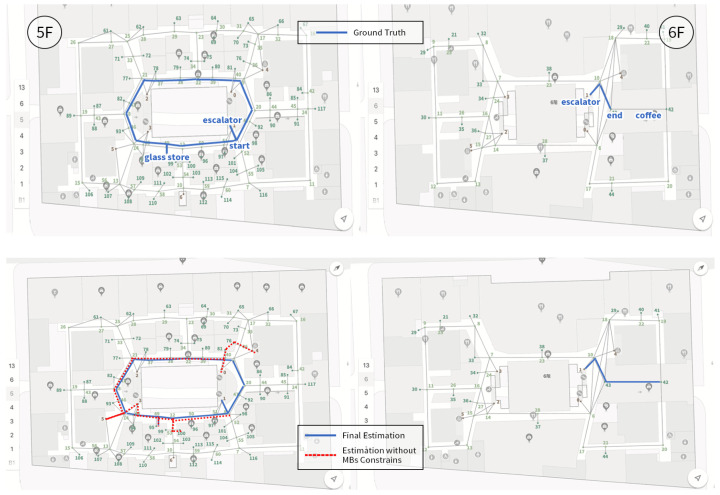
Trajectory 1 of CCA estimation.

**Figure 10:**
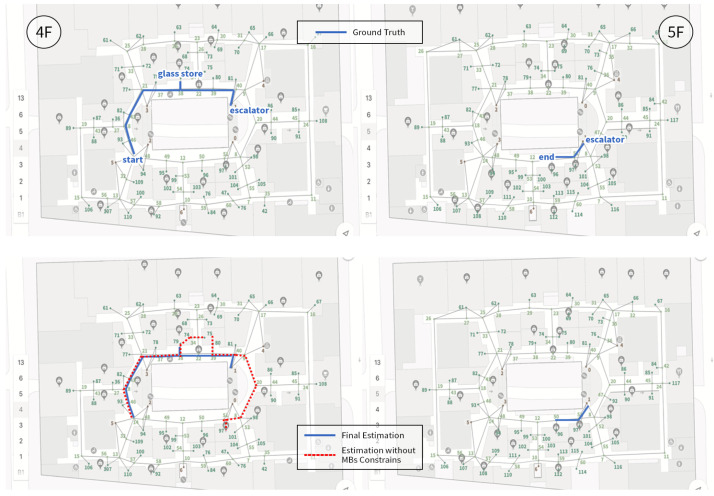
Trajectory 2 of CCA estimation.

**Figure 11:**
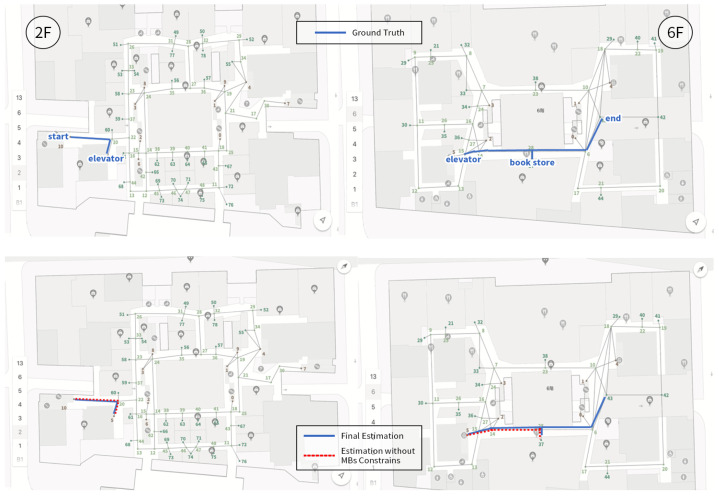
Trajectory 3 of CCA estimation.

**Figure 12:**
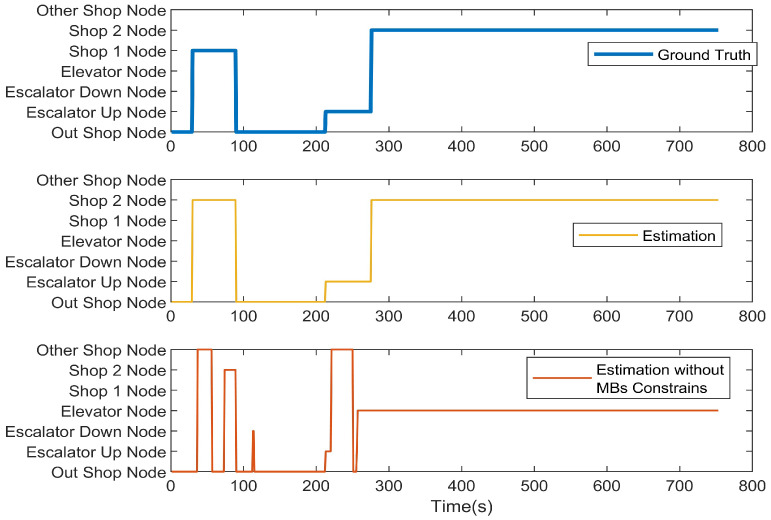
Node estimation of Trajectory 1.

**Figure 13:**
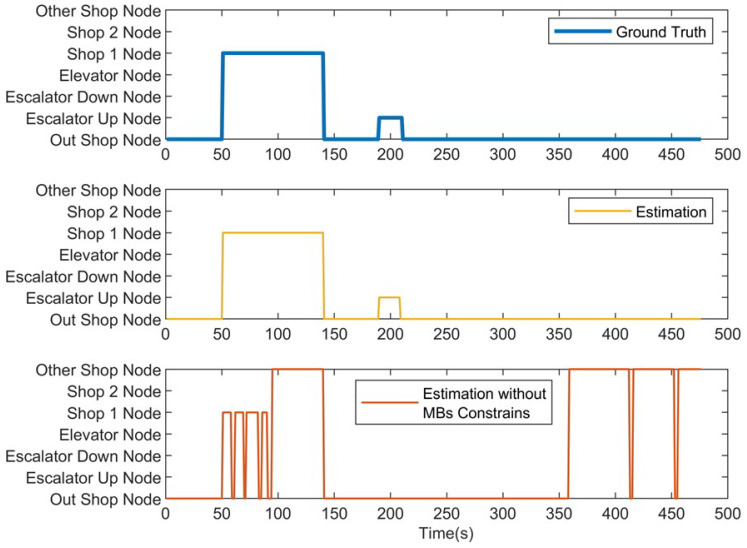
Node estimation of Trajectory 2.

**Figure 14:**
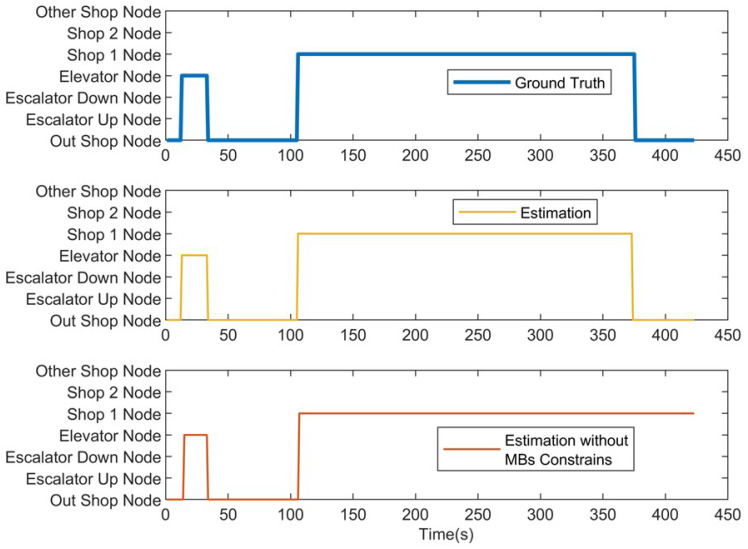
Node estimation of Trajectory 3.

We first present the CCA sequence estimation results based solely on SB recognition. As seen in the real-world test cases, due to the inherent freedom and diversity of human behavior, along with the performance limitations of the model, misclassification issues are inevitable Figure 6, Figure 7, Figure 8, Figure 9, Figure 10, Figure 11, Figure 12, Figure 13 and Figure 14.

This figure illustrates that certain behaviors are prone to confusion during the evaluation of actual behavior sequences. We attempt to analyze the reasons behind these confusions. For example, in Trajectories 1 (Figure 6, Figure 9 and Figure 12) and 3 (Figure 8, Figure 11 and Figure 14), the action of “select items” is often confused with “walking”. This is because, when customers are selecting items, the phone is typically not held in their hands, making it difficult for the phone to directly measure hand movements. Therefore, our definition of “selecting an item” is primarily based on complex movement patterns. We assume that customers exhibit ambiguous, slow, frequent stopping, and turning “loitering” behaviors when selecting items. However, when customers show only brief interest in an item, their behavior pattern closely resembles regular walking.

In Trajectory 2 (Figure 7, Figure 10 and Figure 13), “wait in queue” is sometimes misclassified as “sit” or “select items”. If magnetic interference is introduced in the surrounding environment, it may even be confused with “elevator”. This ambiguity largely arises from the inherent nature of queuing. Within a limited time window, queuing may appear nearly stationary, making it almost indistinguishable. Similarly, selecting items involves frequent stopping as a subbehavior. Therefore, when queuing involves repeated forward/stop patterns within a time window, it is prone to being misclassified as “select items”.

Additionally, in Trajectories 2 (Figure 7, Figure 10 and Figure 13) and 3 (Figure 8, Figure 11 and Figure 14), we observed occasional confusion between “escalator up” and “escalator down” while on an escalator. This is because, during stable escalator operation, no detectable acceleration is present, making it challenging to determine the movement direction. In contrast, within an elevator, the sensation of weightlessness during ascent and increased weight during descent is distinct, which is reflected in improved classification performance. This unique physical feedback aids in more accurately distinguishing upward and downward movements, enhancing the model’s ability to correctly identify these behaviors. According to Equation (6), the behavior sequence estimation accuracy for the three trajectories is as follows: Traj1: 85.66%, Traj2: 84.11%, Traj3: 90.54%.

## 6. Discussion

In this section, we aim to discuss the current limitations of this framework and the insights it provides for future research.

### 6.1. Further Technical Improvements

Our technical framework is based on deeply mining the time-frequency spectrum information of sensor data, which generally makes our method superior to other time-series-based methods. Similarly, we explored the long-range interactions of time-frequency bands and the sequential characteristics in the frequency domain, which makes our approach also outperform recent time-frequency-spectrum-based methods. In our experiments, we found that the recognition rates for simple mobility-related behaviors are usually higher than those for complex semantic activities. A possible reason is that our dataset was collected in the real world, inevitably introducing more noise unrelated to the behavior definitions themselves, such as individual behavioral habits and random body movements. This noise affects simple activities and complex activities differently. For simple activities, the distinguishing criterion could be a significant subaction, such as lifting or placing a leg for walking. In contrast, the judgment criteria for complex activities involve a combination of multiple subactions, and the introduction of noise significantly disrupts this combination. To address this issue, one potential technical improvement is compressed sensing, mapping features to a lower-dimensional latent space to guide the model in learning more salient features and reducing the impact of noise. This will be one of our future research directions.

### 6.2. Practical Application

We discuss the challenges our framework must address when transitioning to real-world applications. These challenges primarily include ensuring robustness during deployment, as well as potential integration methods and privacy concerns when implementing the framework.

In practical deployment, integration methods must also be addressed. Typically, due to issues with parameter size and inference speed, deep learning models are difficult to deploy directly on mobile devices. Since our method is based on time-frequency spectrum data, the parameter size and inference complexity are relatively greater than models based on time-series data. However, the advent of cloud technology reduces deployment difficulties, as computational speed can be offset by data transmission speed. However, this inevitably brings privacy concerns.

To address privacy issues in cloud technology, sensitive data should be encrypted during transmission and storage, with robust access controls and multi-factor authentication in place. Data anonymization and de-identification can minimize exposure risks, while compliance with regulations such as GDPR ensures legal protection. Transparency in data policies, regular audits, and choosing certified providers further enhance trust. Privacy-enhancing technologies like differential privacy and homomorphic encryption, combined with distributed storage and edge computing, reduce centralized vulnerabilities. Additionally, user awareness and adherence to best practices are crucial for comprehensive privacy protection.

### 6.3. Generalization

In this paper, we proposed a simultaneous localization and behavior recognition framework and conducted a case study in a typical large offline shopping mall. In this case study, we defined a set of fundamental and common shopping behaviors observed in large malls. However, in practical applications, it is necessary to define additional specific shopping behaviors to provide sufficient semantic information for different types of retail environments. For instance, in a clothing-focused shopping mall, behaviors such as trying on clothes should be defined to better assess customers’ purchasing intentions.

Our framework can be adapted to different types of shopping venues by extending the dataset, including defining distinct behaviors for various mall types and creating more detailed semantic maps, such as specifying the type of each store. This serves as an inspiration for our future research. We plan to further expand our dataset in terms of both scale and semantic richness.

## 7. Conclusions

In this paper, we propose a novel framework for consumer context analysis to enable the sequential analysis of consumer behaviors in large offline shopping malls. The behavioral semantics of interest include consumers’ movement trajectories within the mall, purchasing behaviors in stores, and dwell times at specific locations. This framework is based on deeply integrated behavior recognition and indoor localization technologies, leveraging short time-window data to estimate fundamental movement behaviors and walking speed in parallel. By employing PDR and map-matching techniques, the framework utilizes speed and node maps to infer the semantic locations of consumers within the shopping mall. Subsequently, complex semantic behaviors are estimated based on long-time-window data, and the final consumer behavior estimation sequence is generated by integrating these behaviors with user location data.

We constructed a dataset in a large shopping mall located in Tokyo and conducted extensive experiments. First, we evaluated the performance of consumer speed estimation on both the Tokyo dataset and the publicly available RIDI dataset, demonstrating that our method achieves lower speed estimation errors and trajectory relative errors. Furthermore, experiments on consumer behavior sequence analysis using the Tokyo dataset showed that our method achieved over 90% accuracy in consumer behavior estimation across the time series.

## Figures and Tables

**Table 1:** Definition of customer behavior and context.

MB	SB	EC
Walk Forward	Select Items	In Shop
Walk Laterally	Wait in Queue	
Still		
Turn	Floor Change (Elevator)	At Elevator/Escalator Node
	Floor Change (Escalator)	
	Sit	Wherever
	Smartphone on Table	

**Table 2:** Parameter setting of CCA.

Layer Name	Parameter
Window Length	SBs:12s, MBs:1s
Wavelet	′*cmor*3 − 3′
Wavelet Scale	64
CNN Layer 1	Res2d[(3,1,64),(3,1,64),(3,2,64)]
CNN Layer 2	Res2d[(3,1,128),(3,1,128),(3,2,128)]
CNN Layer 3	Res2d[(3,1,256),(3,1,256),(3,2,256)]
CNN Layer 4	Res2d[(3,1,512),(3,1,512),(3,2,512)]
LSTM Layer	LSTM(512, 1)
Channel-Attention	ECA(3)
Self-Attention	Att(4, 20, 8, 12)
Active Function	Relu

Res2d means the 2D residual convolutional block, Res[(a,b,c)] means the convolution with the kernel size of *a*, stride of *b*, and the channel size is *c*. LSTM(d,e) means LSTM layer with hidden size of *d* and the layer number is *e*. ECA(h) means ECA-net [35] with kernel size of *h*, Att(i∗j,k,l) means the Self-Attention framework [36] with patch size of i∗j, head number of *k*, and layer number of *l*.

**Table 3:** Parameter settings of the Tokyo dataset.

	Behavior Recognition Set	Velocity Estimation Set
Data length (s)	11,744	6376
Data Split	Train: 80%, Vali: 20%	Train: 80%, Vali: 20%
Sampling Rate (Hz)	Acc, Gyro, Magn: 100. Pres: 1	Acc, Gyro: 100. GPS: 1
Ground Truth	Self-Reported	GPS
Device	iPhone 13	iPhone 13

**Table 4:** Performance of velocity estimation on two datasets.

	Tokyo-Vali		RIDI	
Methods	MSE	RDE	MSE	RDE
Proposed	0.33 m/s	0.8%	0.08 m/s	0.74%
Proposed no-MBs	0.35 m/s	3.6%	0.12 m/s	2.7%
DAE	0.34 m/s	1.7%	0.09 m/s	1.16%
PDR-Net	0.36 m/s	6.2%	0.20 m/s	3.1%
Weinberg	0.51 m/s	18.7%	0.37 m/s	14.3%

**Table 5:** SBs’ recognition performance on the Tokyo dataset.

F1-Score/%	Sit	Walk	Table	Escalator Up	Escalator Down	Elevator Up	Elevator Down	Select Items	Wait in Queue	Total
Proposed	**90.31**	89.21	**99.98**	**90.54**	**91.83**	**94.37**	**92.63**	**93.44**	**87.77**	**92.57**
TNP	72.74	79.11	99.95	65.85	56.31	53.21	47.33	70.01	69.53	64.90
MSRLSTM	80.21	88.93	99.98	76.34	83.94	73.36	77.34	88.65	72.33	81.16
DCL	79.34	80.63	99.76	81.35	87.71	72.46	71.35	83.54	71.64	80.03
ABLSTM	70.44	79.01	99.88	59.35	61.27	66.03	67.89	87.40	70.43	72.57
PEN	79.37	86.81	99.83	89.64	72.56	69.24	70.69	85.11	79.23	81.47
PCL	85.74	**89.72**	99.88	78.22	89.27	86.73	87.41	89.40	79.49	87.69

## Data Availability

The dataset could be available on request.
